# Dataset on aqueous solid-liquid extraction of gossypol from defatted cottonseed in acidic medium using green solvent, its kinetics and thermodynamics study and mass transfer effects

**DOI:** 10.1016/j.dib.2020.105620

**Published:** 2020-04-27

**Authors:** Surinder Singh, Surendra Kumar Sharma, Sushil Kumar Kansal

**Affiliations:** a Dr. S.S. Bhatnagar University Institute of Chemical Engineering & Technology, Panjab University, Chandigarh-160014, India; b University School of Chemical Technology, Guru Gobind Singh Indraprastha University, Sec-16-C, Dwarka-110078, Delhi, India

**Keywords:** Cottonseed, Ethanol, Solvent extraction, Gossypol, Green solvent, Kinetics, Mass transfer

## Abstract

Extraction of gossypol from cottonseed is essentially required to produce cottonseed free from gossypol for animal feed and or human applications. The focus of the present research was to determine the percentage gossypol extraction after extracting the defatted cottonseed using environment friendly green solvent ethanol-water (95:5 v/v) acidified with 0.5 M oxalic acid. The cottonseed samples were taken according to the fixed solvent to seed ratio and were extracted in batch process using round bottom flasks maintained at required temperatures for different extraction times ranging from 5 to 180 mins. After extraction the samples were filtered and dried and subjected to total gossypol analysis using BIS method. One factor at a time (OFAT) experimental design was employed to optimize the different process parameters like acid type and concentration, solvent to seed ratio, temperature and contact time. The obtained data was studied for analysis of kinetics of extraction using three different kinetic models, calculation of activation energy, evaluating values of kinetic parameters and thermodynamic parameters. The data was also analyzed for evaluation of mass transfer effects viz. liquid film diffusion and internal solid diffusion and calculation of diffusion rate constants for the extraction of gossypol from cottonseed. The present dataset demonstrated the analysis of experimental data for determining the type of kinetics, thermodynamic parameters and mass transfer effects of the solvent extraction for future researchers.

Specifications Table

**Table:** 

Subject	Filtration and Separation
Specific subject area	Chemical Engineering-Separation Technology
Type of data	Table and Figure
How data were acquired	UV-Vis Double beam Spectrophotometer ( Systronic, model 2202)
Data format	Raw, analysed
Parameters for experimental data collection	The data was experimentally obtained at fixed pressure of 0.974 bar (731 mm Hg) and at respective temperatures as mentioned in the text i.e. figures and tables etc.
Description of Experimental Data Collection	The experimental data for solvent extraction was obtained using batch extraction and total gossypol was analysed using UV-Vis spectrophotometer using standard BIS method.
Data source location	Chandigarh-160014, India.
Data accessibility	Raw data is given in this article.

## Value of the data

• Kinetics and mass transfer analysis is essential for any solid-liquid extraction process. This data provides methodology to apply kinetic models and their applicability in extraction, gives evaluation and effect of diffusion rate constants and effect of process parameters on kinetics.
• This data can be used by researchers/scientists/investigators who work in the field of solid-liquid extraction and separation science and/or technology.
• The mass transfer effect on extraction and evaluation of thermodynamic parameters has been elucidated in simple and descriptive manner which will be useful for all the fellow researchers.
• This dataset can be used as a tool to identify the kinetics of extraction and mechanisms affecting the solid-liquid extraction process.

## 1 Data Description

**Fig. 1:**
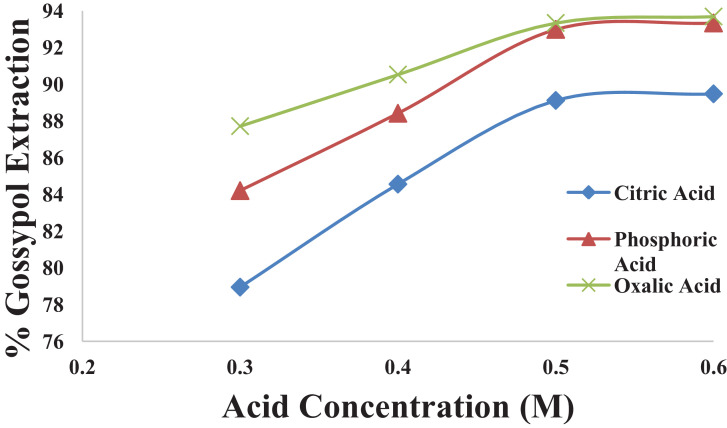
Effect of acid concentration on gossypol extraction at 348 K, SR15 and 180 min.

**Fig. 2:**
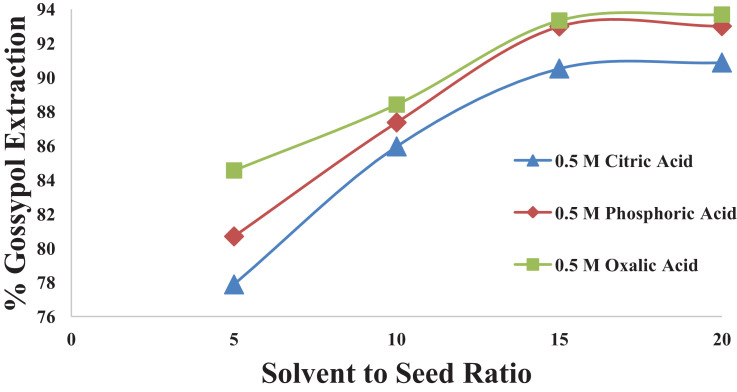
Effect of solvent to seed ratio using different acids (180 min, 348 K)

**Fig. 3:**
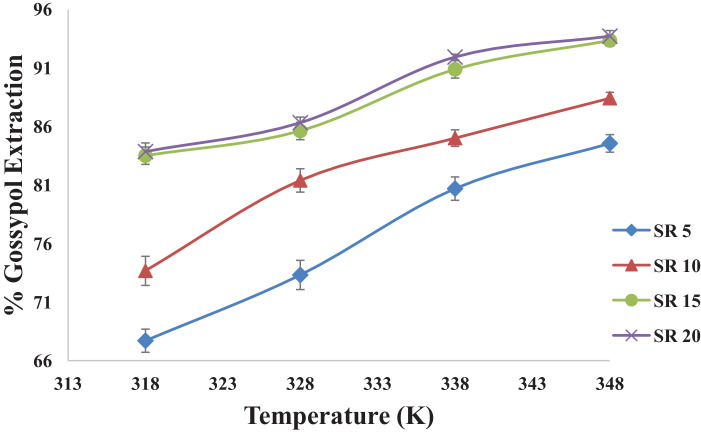
Effect of temperature on gossypol extraction at different solvent to seed ratios using ethanol-water solvent acidified with 0.5 M oxalic acid in 180 min.

**Fig. 4:**
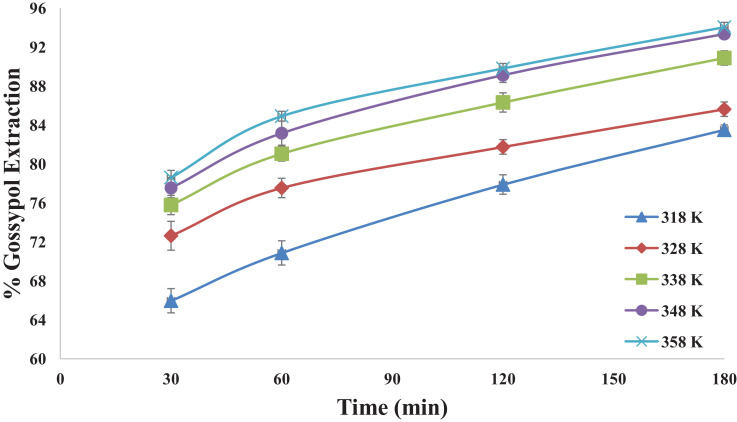
Effect of time at different temperatures on extraction using Ethanol-water solvent acidified with 0.5 M oxalic acid at SR15 and 180 min

This dataset contains 12 Figures and 13 tables that represent the solid-liquid extraction data, kinetics of extraction, thermodynamics and diffusion rate constants for the extraction of gossypol from defatted cottonsed using ethanol-water (95:5v/v) solvent. One factor at a time (OFAT) experimental design was used to optimized the process parameters. Fig. 1 shows the effect of acid concentartion on gossypol extarction using three different acids at 348 K and solvent to seed ratio (SR) of 15 in 180 min. Fig. 2 shows the effect of solvent to seed ratio on gossypol extraction using three different acids at 0.5 M conc., 348 K temeperature and in extraction time of 180 mins. Fig. 3 shows the effect of temperature on gossypol extraction at different solvent to seed ratios. Fig. 4 shows effect of time at different temperatures on extraction at SR15 and 180 min. The average % error was found out to be 1.4%. The optimum conditions obtained were 0.5 M oxalic acid, SR 15, 348 K temperature and 180 min contact time.

**Fig. 5:**
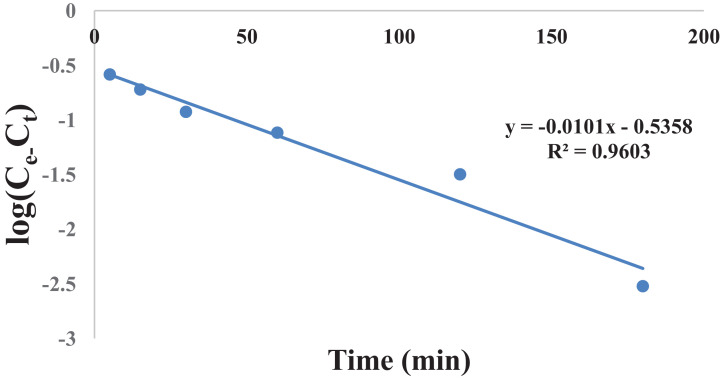
Pseudo first order kinetics of gossypol extraction at SR 15 and 348 K

Table 1, Table 2, Table 3, Table 4 correspond to raw data of Fig. 1 to Fig. 4 Fig. 5 shows the fitting of experimental data into pseudo first order kinetic model for gossypol extraction at SR 15 and 348 K. Table 5 gives the raw data for Fig. 5.

**Table 1:** Effect of acid concentration on gossypol extraction at 348 K, SR15 and 180 min.

S. No.	Acid Concentration(M)	% Gossypol Extraction		
		Citric Acid	Phosphoric acid	Oxalic acid
1	0.3	78.94	84.2	87.71
2	0.4	84.55	88.41	90.52
3	0.5	89.11	92.98	93.33
4	0.6	89.47	93.33	93.68

**Table 2:** Effect of solvent to seed ratio on gossypol extraction at 348 K, SR15 and 180 min.

S. No.	Solvent to Seed Ratio (SR)	% Gossypol Extraction		
		Citric Acid	Phosphoric acid	Oxalic acid
1	5	77.88	80.69	84.55
2	10	85.96	87.36	88.41
3	15	90.52	92.98	93.33
4	20	90.87	93	93.68

**Table 3:** Effect of temperature on gossypol extraction at different solvent to seed ratios with Ethanol-water acidified with 0.5 M oxalic acid in 180 min

S. No.	Temperature (K)	% Gossypol Extraction (mean values)			
		SR-5	SR-10	SR-15	SR-20
1	318	67.71	73.67	83.5	83.85
2	328	73.32	81.39	85.61	86.31
3	338	80.69	85.00	90.87	91.92
4	348	84.55	88.41	93.33	93.7

**Table 4:** Effect of time at different temperatures on extraction at SR15 and 180 min

S. No.	Time (min)	% Gossypol Extraction with Temperature (K), mean values				
		318 K	328 K	338 K	348 K	358 K
1	30	65.95	72.62	75.78	77.53	78.6
2	60	70.86	77.53	81.04	83.15	84.9
3	120	77.88	81.74	86.31	89.11	89.8
4	180	83.5	85.61	90.87	93.33	94.03

**Table 5:** Pseudo first order kinetics data for gossypol extraction at 348 K and SR15

S. No.	Time (min)	Log (C_e_-C_t_)
1	5	−0.58486
2	15	−0.72285
3	30	−0.92702
4	60	−1.11748
5	120	−1.49894
6	180	−2.52288

**Fig. 6:**
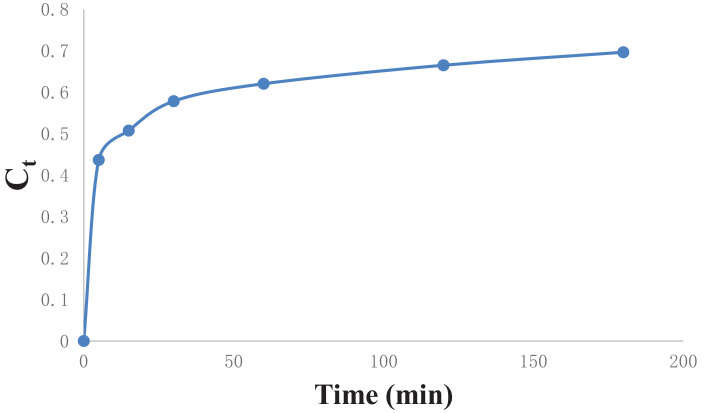
Plot of extraction of gossypol (C_t_) vs time at 348 K at SR 15 for Ethanol-water (95:5)-0.5 M oxalic acid

**Fig. 7:**
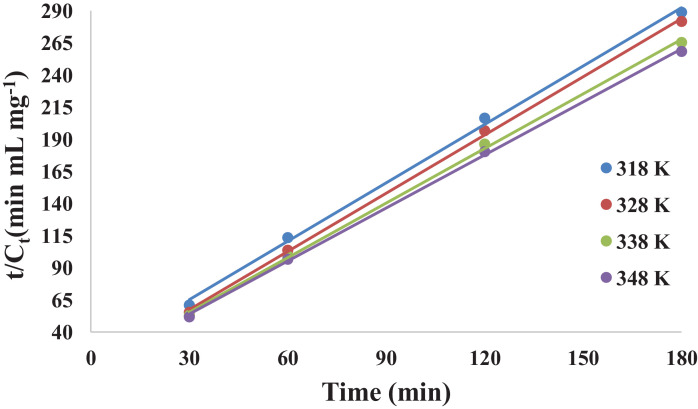
Pseudo second order kinetics of gossypol extraction at different temperatures (t/C_t_ vs time) for Ethanol-water (95:5)-0.5 M oxalic acid at SR15

**Fig. 8:**
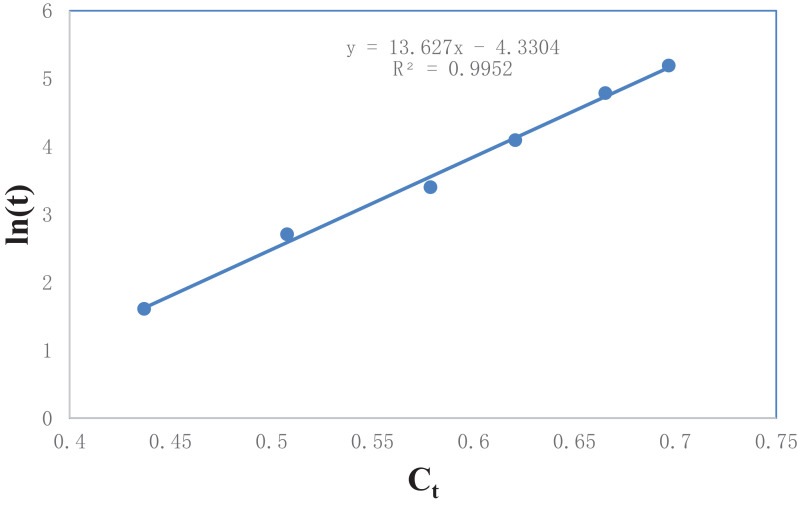
Elovich kinetics model for kinetics of gossypol extraction at 348 K (ln(t) vs Ct) for Ethanol-water (95:5)-0.5 M oxalic acid

**Fig. 9:**
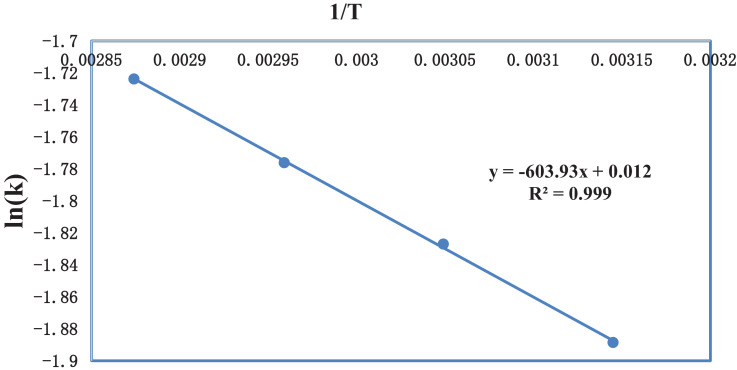
Plot of ln (k) vs 1/T

**Fig. 10:**
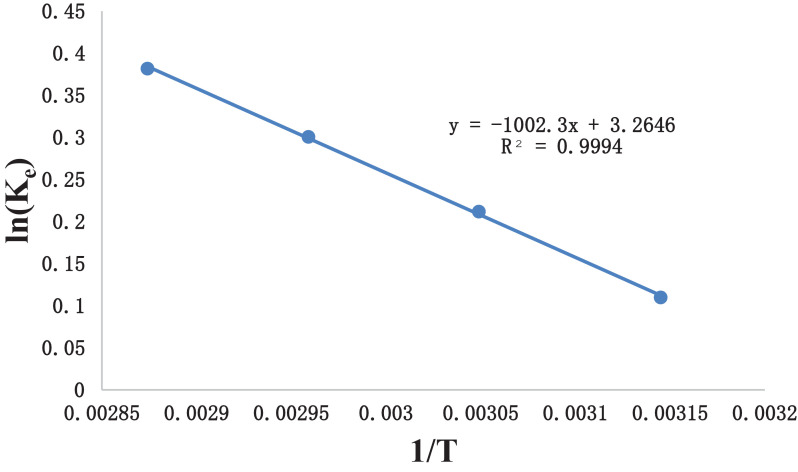
Plot of ln (K_e_) vs 1/T for gossypol extraction (Vant Hoff's plot)

**Fig. 11:**
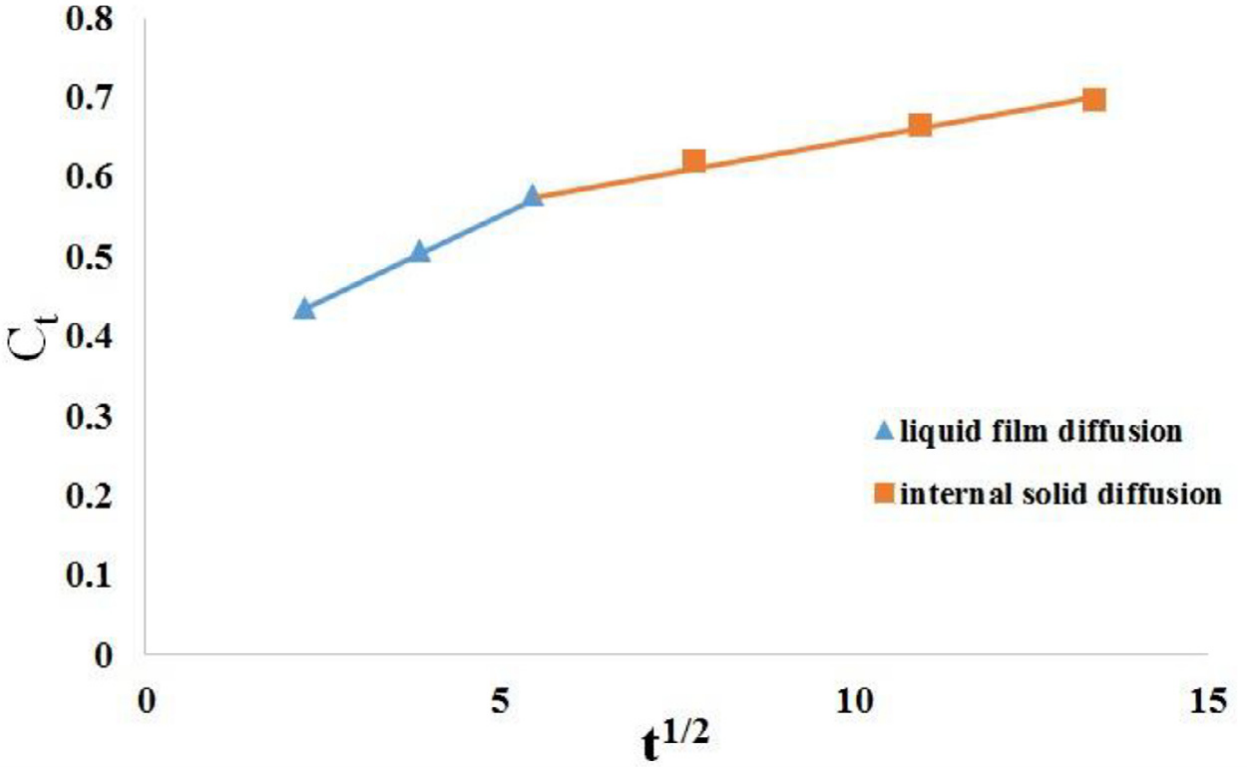
Plot of **C_t_** vs **t^1/2^** for Ethanol-water system

**Fig. 12:**
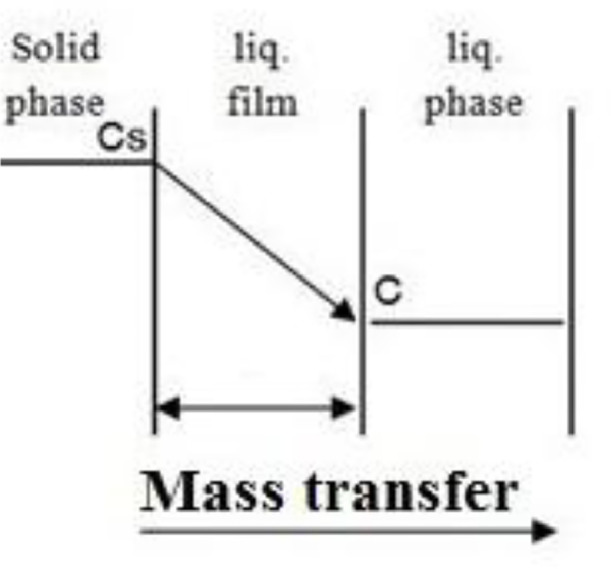
Mass transfer effect during solid-liquid extraction

Table 6 gives the values of evaluated first order rate constant and extraction capacity at saturation. Fig. 6 shows the graph of extraction of gossypol (C_t_) vs time at 348 K at SR 15. Fig. 7 shows the pseudo second order kinetics by plotting a graph between t/C_t_ and time. Tables 7 and 8 correspond to raw data of Fig. 6 and Fig. 7. Table 9. gives the values of kinetic parameters for gossypol extraction for pseudo second order model at SR15 and different temperatures. Fig. 8 describes the Elovich kinetics model and Table 10 corresponds to raw data of Fig. 8. Table 11 gives the values of Elovich kinetic model constants. Fig. 9 shows the plot of ln (k) vs 1/T to evaluate activation energy. Fig. 10 describes the Vantt Hoff's plot i.e. ln (K_e_) vs 1/T for evaluating thermodynamic parameters for gossypol extraction. The raw data for Fig. 9 (value of k) and Fig. 10 (value of Ke) had been taken from Tables 9 and 12 and used after taking natural logarithm of k and K_e_ values respectively. Table 12 gives values of thermodynamic parameters for gossypol extraction. Fig. 11 describes mass transfer effect i.e. intraparticle diffusion model depicting plot of C_t_ vs t^1/2^. Fig. 12 explains the mass transfer mechanism of solid-liquid extraction. The raw data for Fig. 11 had been used from Table 7. Table 13 gives the values of diffusion rate constants i.e. liquid film diffusion constant and internal solid diffusion constant (Table 8).

**Table 6:** Rate constant (k) and extraction capacity (C_e_) using pseudo first order model

SR, mL/g	Temp, K	Slope (x 10^2^)	k (min^-1^)	Intercept	Ce (mg mL^-1^)	R^2^
15	348	−1.01	0.0233	−0.5358	0.2912	0.9603

**Table 7:** Plot of extraction of gossypol (C_t_) vs time at 348 K at SR 15

S. No.	Time (min)	C_t_
1	0	0
2	5	0.4369
3	15	0.5077
4	30	0.5787
5	60	0.6207
6	120	0.6653
7	180	0.6967

**Table 8:** Pseudo second order kinetics of gossypol extraction at different temperatures (t/C_t_ vs time)

S. No.	Time (min)	t/C_t_ (min mL mg^-1^)			
		318 K	328 K	338 K	348 K
1	30	60.97	55.36	53.04	51.84
2	60	113.47	103.68	99.19	96.67
3	120	206.44	196.67	186.25	180.37
4	180	288.79	281.67	265.32	258.33

**Table 9:** Kinetic parameters for gossypol extraction for pseudo second order model at SR15

Temp, K	E_i_ (mg mL^-1^min^-1^)	k(mLmg^-1^min^-1^)	C_e_ (mg/mL^-1^)	R^2^
318	0.0630	0.1513	0.6329	0.9996
328	0.0752	0.1609	0.6711	0.9993
338	0.0824	0.1694	0.6976	0.9997
348	0.0893	0.1784	0.7155	0.9998

**Table 10:** Elovich kinetics plot (ln(t) vs Ct) for kinetics of gossypol extraction at 348 K

S. No.	C_t_	Ln (t)
1	0.4369	1.609438
2	0.5077	2.70805
3	0.5787	3.401197
4	0.6207	4.094345
5	0.6653	4.787492
6	0.6967	5.192957

**Table 11:** Elovich kinetic model constants

SR, mL/g	Temp, K	Slope	β (mLmg^-1^)	Intercept	α (mg mL^-1^min^-1^)	R^2^
15	348	13.62	0.0734	−4.3304	9.917	0.9952

**Table 12:** Thermodynamic parameters for extraction of gossypol employing Ethanol-water (95:5 v/v) solvent acidified with 0.5 M oxalic acid at SR 15.

Temperature K	Equilibrium constant (Ke)	Gibbs free energy (ΔG^o^) J/mol	ΔH^o^ J/mol	ΔS^o^ J/mol K
318	1.1160	−290.031	8333.122	27.14
328	1.2358	−577.304		
338	1.3505	−844.445		
348	1.4647	−1104.07

**Table 13:** Mass transfer model rate constants for gossypol extraction

Gossypol extraction; **SR** (at 348 K)	Diffusion rate constants, (mg/ml•min^0.5^)	
	Liquid film diffusion constant	Internal solid diffusion rate constant
	**K_1_**	**K_2_**
15 mL/g	0.0437	0.0134
	**Intercept on x-axis**	
15 mL/g	0.3388	0.5172

## 2 Experimental Design, Materials, and Methods

### 2.1 Experimental design

One factor at a time (OFAT) experimental design was used in this work to optimize the process parameters i.e. acid type and concentration, solvent to seed ratio, temperature and contact time. The obtained experimental data was analysed to determine the kinetics of extraction, thermodynamic parameters and mass transfer effect.

### 2.2 Materials

Cotton variety used was RCH-776- BT cotton (*G. Hirsutum*) hybrid variety procured from local market. Ethanol, 3-amino-1-propanol, glacial acetic acid, oxalic acid, citric acid, phosphoric acid and N, N dimethyl formamide were purchased from Merck Specialities Private Ltd, India. All stock solutions were prepared using double distilled water made in laboratory. Gossypol standard was purchased from Sigma Aldrich, India. All chemicals used were of analytical grade.

### 2.3 Gossypol extraction procedure

Known amount of defatted cottonseed sample with ethanol-water (95:5 v/v) solvent at desired solvent to seed ratio was taken in a flat bottom flask. The mixture was extracted at desired temperature (318, 328, 338 and 348 K) using temperature controlled hot plate kept in glass (closed) enclosure, a stir bar (250 rpm) was utilized for proper contact. After known periods of extraction time the sample was filtered using buchner funnel. The filtered sample was then dried at a temperature of 50 ^0^C using a convection oven for 12 hours. The dried sample was then analysed for total gossypol using UV-Vis Double beam Spectrophotometer.

### 2.4 Analysis of Total Gossypol

The analysis of total gossypol content was determined using UV-Vis Double beam Spectrophotometer as per BIS standard method IS: 4876-1986 [1,2]. The percentage gossypol extraction was calculated from total gossypol content of the sample extracted and initial seed.

### 2.5 Kinetics and thermodynamics of gossypol extraction

The kinetics of gossypol extraction was analysed using three different models namely pseudo first order model, Elovich model and pseudo second order model as thoroughly explained by H.A. Harouna-Oumarou et al., 2007 [3] and other reserachers [2], [3], [4], [5], [6], [7], [8]. The thermodynamic parameters were evaluated as discussed by Singh et al., 2019 [2] and others [6,[9], [10]

### 2.6 Green solvent

Ethanol–water was chosen as green solvent as it is environment friendly and also ethanol qualifies as a green solvent as per CHEM21 solvent selection guide [11].

The values of activation energy, E and specific rate constant, k_0_ were calculated from the slope and intercept of graphical plot between ln (k) and 1/T as shown in Fig. 9 [2]. The obtained values of k_0_ and E were 0.012 mLg^-1^min^-1^ and 5.021 kJ mol^-1^ respectively.

### 2.7 Mass transfer effects

The effect of mass transfer on solid-liquid extraction of gossypol was analysed using intra-particle diffusion model [12] and mechanism of solid-liquid extraction as per discussed by Harouna-Oumarou et al., 2007 [3].

#### 2.7.1 Mass transfer model

To study the effect of mass transfer on solid liquid extraction kinetics of Ethanol-water system a graph of C_t_ vs t^1/2^ (intra-particle diffusion model) at optimum conditions was plotted as shown in Fig. 11.

The first part of the curve is attributed to liquid film effect i.e. liquid film diffusion (slope K_1_) taking place at solid-liquid interface, while the second linear part indicates internal solid diffusion (slope K_2_). The diffusion rate parameters K_1_ and K_2_ as obtained are shown in Table 5. The diffusion rate parameters indicate that the internal solid diffusion controls the extraction rate; which is the slowest step in extraction.

## Appendix Supplementary materials

Image, application 1
